# Vocal usage learning and vocal comprehension learning in harbor seals

**DOI:** 10.1186/s12868-024-00899-4

**Published:** 2024-10-04

**Authors:** Diandra Duengen, Yannick Jadoul, Andrea Ravignani

**Affiliations:** 1 Comparative Bioacoustics Research Group, Max Planck Institute for Psycholinguistics, Nijmegen, The Netherlands; 2 Zoo Cleves (“Tiergarten Kleve”), 47533 Kleve, Germany; 3 Center for Music in the Brain, Department of Clinical Medicine, Aarhus University & The Royal Academy of Music, Aarhus, Denmark; 4 Department of Human Neurosciences, Sapienza University of Rome, Rome, Italy

**Keywords:** Vocal learning, Playback study, Double-blind study, Auditory generalization, Auditory discrimination, *Phoca vitulina*, Pinniped

## Abstract

**Background:**

Which mammals show vocal learning abilities, e.g., can learn new sounds, or learn to use sounds in new contexts? Vocal usage and comprehension learning are submodules of vocal learning. Specifically, vocal usage learning is the ability to learn to use a vocalization in a new context; vocal comprehension learning is the ability to comprehend a vocalization in a new context. Among mammals, harbor seals (*Phoca vitulina*) are good candidates to investigate vocal learning. Here, we test whether harbor seals are capable of vocal usage and comprehension learning.

**Results:**

We trained two harbor seals to (i) switch contexts from a visual to an auditory cue. In particular, the seals first produced two vocalization types in response to two hand signs; they then transitioned to producing these two vocalization types upon the presentation of two distinct sets of playbacks of their own vocalizations. We then (ii) exposed the seals to a combination of trained and novel vocalization stimuli. In a final experiment, (iii) we broadcasted only novel vocalizations of the two vocalization types to test whether seals could generalize from the trained set of stimuli to only novel items of a given vocal category. Both seals learned all tasks and took ≤ 16 sessions to succeed across all experiments. In particular, the seals showed contextual learning through switching the context from former visual to novel auditory cues, vocal matching and generalization. Finally, by responding to the played-back vocalizations with distinct vocalizations, the animals showed vocal comprehension learning.

**Conclusions:**

It has been suggested that harbor seals are vocal learners; however, to date, these observations had not been confirmed in controlled experiments. Here, through three experiments, we could show that harbor seals are capable of both vocal usage and comprehension learning.

**Supplementary Information:**

The online version contains supplementary material available at 10.1186/s12868-024-00899-4.

## Background

Vocal learning comprises *contextual learning* and *production learning*, both of which rely on experience [1]. Vocal production learners can modify innate vocalizations or innovate/imitate novel ones [2]. Vocal contextual learning manifests in two different forms: Understanding that an innate or learned vocalization has a different function depending on context is classically termed *comprehension learning* while using a vocalization in a novel context is termed *usage learning* [1, 3]. Learning to vocalize in the presence of an arbitrary stimulus (e.g., a hand sign) is an example of vocal usage learning; instead, comprehension learning requires the association of a vocalization with an action (i.e., a response) [4].

Vocal learning is a building block of human speech, which in turn requires the ability to learn, produce, and interpret complex signals, depending on context and experience [5]. The comparative approach, investigating related abilities in related species, can provide insight into the evolution of speech and vocal learning in general. Vocal learning is much researched in birds, especially songbirds and parrots. Both African grey parrots (*Psittacus erithacus*) (for a review see Pepperberg 2010) [7] and budgerigars (*Melopsittacus undulatus*) [8–9] are highly skilled vocal learners who demonstrated their abilities for vocal production and contextual learning in a series of operant conditioning studies. In mammals, vocal production learning is much rarer than in bird species, but contextual learning occurs in a wider range of animal species [1, 10]. Vervet monkeys (*Chlorocebus pygerythrus*) learn to use acoustically different calls depending on the type of predator [11] or individual, as is the case in dolphin (*Tursiops truncatus*) signature whistles [12, 13]. The latter is further a remarkable example of vocal production learning, as signature whistles develop through experience [14]. Vocal usage learning was shown in e.g., bats [e.g., *Phyllostomus discolor*; 15], elephants [e.g., *Loxodonta africana*; 16], toothed whales [e.g., *Delphinapterus leucas*; 17] and passerine birds [e.g., *Dicrurus paradiseus*; 18]. As these examples demonstrate, while vocal learning is rare, it is present in a diverse range of phylogenetically distant species.

An especially good model for the comparative study of vocal learning are phocids (or true seals): Phocids have good articulatory and breathing control and produce some of their vocalizations through their larynx [19–21]. Beyond their comparative and translational value, seals’ vocal learning abilities, both production and contextual, can provide insight into the species’ communication. Two prominent species of investigation are grey seals (*Halichoerus grypus)* [20, 22–25] and harbor seals (*Phoca vitulina)* [21, 26–31]. While no dedicated experiments investigated the extent of this ability in harbor seals (see below), grey seals’ vocal learning abilities were investigated in detail [20, 22–24]. Vocal usage learning can be dissected into at least four levels: calling on cue, calling and refraining from calling on cue, responding to a cue with a specific call from the repertoire, and responding to the playback of an untrained cue with a call of the same class [17, 22]. Two grey seals learned the first three levels of usage learning, but failed at generalizing to novel stimuli, perhaps due to the limited training repertoire in the study. The study was repeated and extended with a female grey seal that was trained to vocally match two different call types [23]. Not only did the seal learn this discrimination task, she also generalized to novel acoustic stimuli. Inspired by these studies, two harbor seals were successfully trained to vocalize and refrain from vocalizing on distinct visual cues and emit two different vocalizations upon presentation of distinct visual cues [32]. However, it remains unclear whether harbor seals are also capable of call matching, generalization, and vocal comprehension learning.

Harbor seals already vocalize during puppyhood; they emit so-called mother attraction calls [33]. Adult males are vocally more active than adult females, which is reflected in their seasonal courtship display [34]. Male harbor seals are hypothesized to “sing songs” [29, 35], which consist of ‘roar’ series and potentially other vocalizations [36], alongside bubble blowing and flipper slapping [37–39]. Adult females instead rarely vocalize, mostly during agonistic interactions [36, 40]. Despite various preceding studies strongly contributing to our understanding of harbor seal vocal communication [34, 36, 39, 40], the extent to which harbor seals can adjust their vocalizations in production and context remains largely unexplored. The ability to discriminate among vocal cues is of ecological importance to many species that rely on vocal communication; the same may hold for the harbor seal, a species that uses not only visual but also acoustic signals for their territorial display.

It is widely assumed that harbor seals are vocal usage and production learners [19, 28, 31, 41]; however, to date, these observations have not been confirmed in controlled experiments. Most of these studies refer to attempts to shape vocalizations through operant conditioning [19, 41], or the imitative abilities of Hoover, a human-raised harbor seal [28, 31, 41]. Indeed, Hoover’s mimicking abilities are unmatched in the non-human mammal world. Hoover’s case is a ‘black boxed’ example of vocal production learning: His vocal behavior has been reported many times [28, 31, 42–44], but the underlying learning mechanisms remain unknown [28, 29]. However, a somewhat detailed case study demonstrates how one seal’s vocalization can be shaped to produce a novel sound upon presentation of a discriminative stimulus, thereby demonstrating usage learning [41]. Despite this being an important first insight, important details of the experimental setup, such as the amount of sessions, the number of trials per session, and the presence of a learning criterion are missing [41]. These details could provide a window into the mechanics of vocal learning.

A popular method to investigate contextual learning is based on operant conditioning [1, 10, 41]. Operant conditioning increases or decreases the frequency of a behavior depending on whether the behavior is reinforced or punished [45]. Pairing a cue, a so-called discriminative stimulus (e.g., a hand gesture), with the desired behavior (e.g., a vocalization) and reinforcing that behavior in the presence of the cue leads to the association between the two. This way, an animal can learn to vocalize in a specific context (e.g., presenting the cue; vocal usage learning) or to respond in a certain way upon presentation of a specific vocalization (e.g., broadcasting the cue; vocal comprehension learning). The ability of harbor seals to vocalize upon presentation of a hand sign has been reported in a case study before [41] and was recently more quantitatively assessed [32]. A more complex contextual learning task than the mere association of one or two vocalizations with a visual cue [32] is that of vocal matching, which requires two different vocalizations and learning to respond in a novel context (i.e., respond to an auditory as opposed to a visual cue) [22, 23]. Here, the animal is asked to respond to the playback of one out of 2 or more vocalizations (cue) with a vocalization of the same type [17, 22]. This approach enables the assessment of both contextual learning submodules: vocal usage and comprehension learning.

To test for auditory generalization and to assess contextual learning in a double-blind study, we tested whether individual harbor seals could switch the context of trained vocal responses [32] from visual to auditory (contextual learning) and if they could generalize from trained to novel auditory stimuli. Auditory generalization occurs when a conditioned behavior (e.g., a vocalization) to a trained stimulus is also elicited by a novel, unconditioned stimulus [46]. This ability is crucial to vocal communication and has been shown in the closely related grey seal [23]. To test for auditory generalization, novel stimuli of the same categories are presented to the animal [22]; these novel stimuli only share some perceptual features with the conditioned stimuli [47].

Through playbacks of harbor seals’ own vocalizations, we tested for the ability of usage and comprehension learning, as well as auditory generalization. In three individual call matching experiments, we tested whether the seals could learn to (i) correctly respond to a trained set of playback stimuli of the vocal types 1 and 2, (ii) correctly respond to a combination of trained and untrained playback stimuli, and (iii) generalize their responses to entirely novel playback stimuli.

## Results

Two seals participated in three experiments testing their abilities for vocal usage and comprehension learning. In *Experiment 1*, the seals were trained to respond to sets of playback stimuli consisting of their own vocalizations. These vocalizations consisted of two types per seal (Type 1 and Type 2), which were termed E1/E2 (seal E), and J1/J2 (seal J), and were previously trained [32]. Subsection ‘Stimulus selection’ in Methods discusses how vocalization types differed from each other. In *Experiment 2*, the seals were exposed to a combination of trained and novel stimuli. In *Experiment 3*, only novel vocalizations of the two vocalization types were played back to test whether the seals could generalize from the trained set of stimuli to only novel items of a given vocal category. The seals’ performance was assessed through a learning criterion (LC) of reaching 80% correct choices in four consecutive sessions in each experiment.

**Fig. 1:**
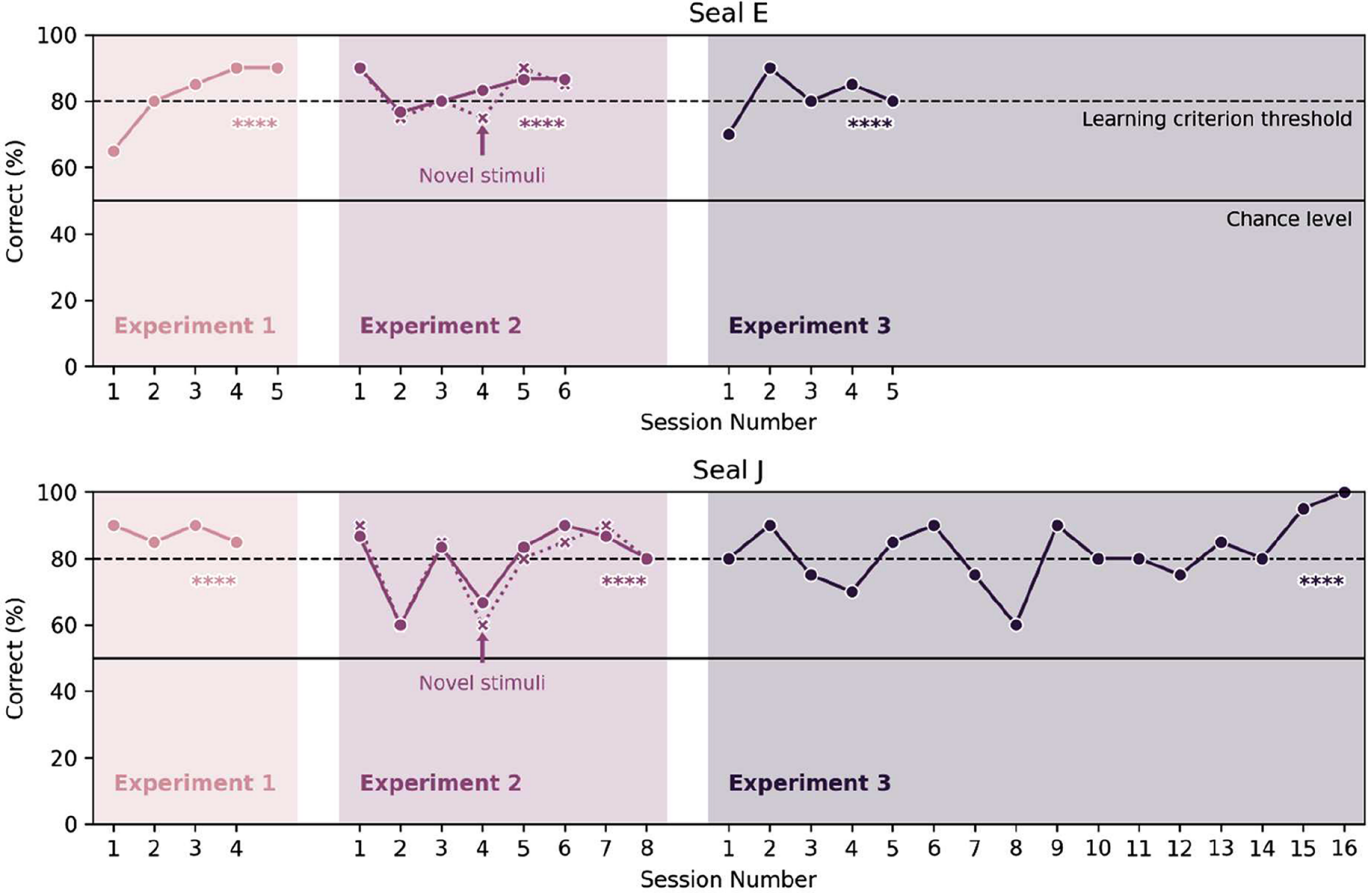
The learning curves of seal E (above) and seal J (below) show that their performance reached the learning criterion (dashed horizontal line) in every experiment. The y-axis depicts the correct choices in % in each session. The solid horizontal line represents the chance level, i.e., the expected performance when randomly guessing. The dotted learning curve in *Experiment 2* depicts the correct responses to only the novel stimuli. The stars (****) indicate a p-value of < 0.0001 of reaching the learning criterion within the observed number of sessions. For a learning curve of the training phase, see Figure S1

Both seals reached the LC in all three experiments in ≤ 16 sessions (Fig. 1). The seals demonstrated both types of vocal contextual learning: usage learning and comprehension learning. This is reflected in the seals’ ability to use different vocalizations accordingly (vocal usage learning) and to appropriately respond to the novel context of their own vocalizations’ playback (vocal comprehension learning). Additionally, they demonstrated the ability for auditory generalization by correctly responding to novel stimuli in the third experiment.

### Experiment 1: discrimination of trained stimuli

In *Experiment 1*, we tested whether the seals could vocally match a set of auditory playback stimuli consisting of their own, previously recorded vocalizations. For this, the seals heard 20 individual stimuli, one per trial, resulting in 20 trials per session (10 per vocalization type, see subsection *Experiment 1* in Methods).

Both seals successfully learned to discriminate the auditory cues and respond to each playback type with the corresponding vocalization type (Fig. 1). Seal E reached the learning criterion within 5 sessions (exact probability calculation, *p* < 0.0001; see Methods), seal J reached the learning criterion within 4 sessions (exact probability calculation, *p* < 0.0001).

### Experiment 2: transition from trained to untrained stimuli

*Experiment 2* served as transition to *Experiment 3*, testing whether the seals had either memorized the priorly learned stimuli individually, or generalized to “types” of vocalizations. Therefore, the seals were introduced to 20 novel stimuli per session (10 per vocalization type), which were sampled from a pool of 200 stimuli that were never played back to them before the start of this experiment (see subsection *Experiment 2*, in the Methods section). Additionally, 10 of the trained stimuli (5 per vocalization type) were randomly interspersed with the new stimuli and played back to the seals, resulting in 30 trials/session.

Both harbor seals could generalize vocalizations according to type (Fig. 1). Seal E reached the learning criterion within 6 sessions (exact probability calculation, *p* < 0.0001), and seal J within 8 sessions (exact probability calculation, *p* < 0.0001).

Seals correctly responded to both *trained* stimuli and novel, untrained stimuli. Seals’ responses were not significantly more correct for either the old or the novel stimuli (seal E: Fisher’s exact test, prior odds ratio = 1.378, *p* > 0.05; seal J: Fisher’s exact test, prior odds ratio = 1.169, *p* > 0.05). Crucially, both seals’ responses to the novel stimuli subset alone were also significantly more often correct than incorrect (seal E: binomial test, *n* = 120, k = 99, *p* < 0.0001; seal J: binomial test, *n* = 160, k = 126, *p* < 0.0001). After having reached the learning criterion, there was a ten-day break from experiments. In order to refresh the experimental task after this break, seal J received four extra sessions with stimuli from *Experiment 2*, before moving on to *Experiment 3*.

### Experiment 3: generalization of novel stimuli

In *Experiment 3*, the seals were asked to respond to entirely novel stimuli of the same vocalization types consisting of 20 trials/session. These stimuli consisted of their own, recorded vocalizations, and had never been played back to them before, nor were they repeated during the experiment. The seals were able to generalize beyond trained (*Experiment 1*) and both trained and untrained (*Experiment 2*) stimuli; seal E reached the LC within 5 sessions (exact probability calculation, *p* < 0.0001), seal J reached the LC within 16 sessions (exact probability calculation, *p* < 0.0001).

### Vocal type preferences

To test whether the seals showed any vocal type preferences that could have had an influence on the experiment and its results, we tested whether they responded with one vocal type more often than the other (i) over all trials, (ii) within correct trials, and (iii) within incorrect trials. None of the seals showed significant response preferences (i) over all trials (seal E: binomial test, *n* = 379, *k* = 195, *p* > 0.05; seal J: binomial test, *n* = 640, *k* = 325, *p* > 0.05), (ii) within correct trials (seal E: binomial test, *n* = 314, *k* = 160, *p* > 0.05; seal J: binomial test, *n* = 523, *k* = 264, *p* > 0.05), or (iii) within incorrect trials (seal E: binomial test, *n* = 65, *k* = 35, *p* > 0.05; seal J: binomial test, *n* = 117, *k* = 61, *p* > 0.05).

### Response parameters

**Fig. 2:**
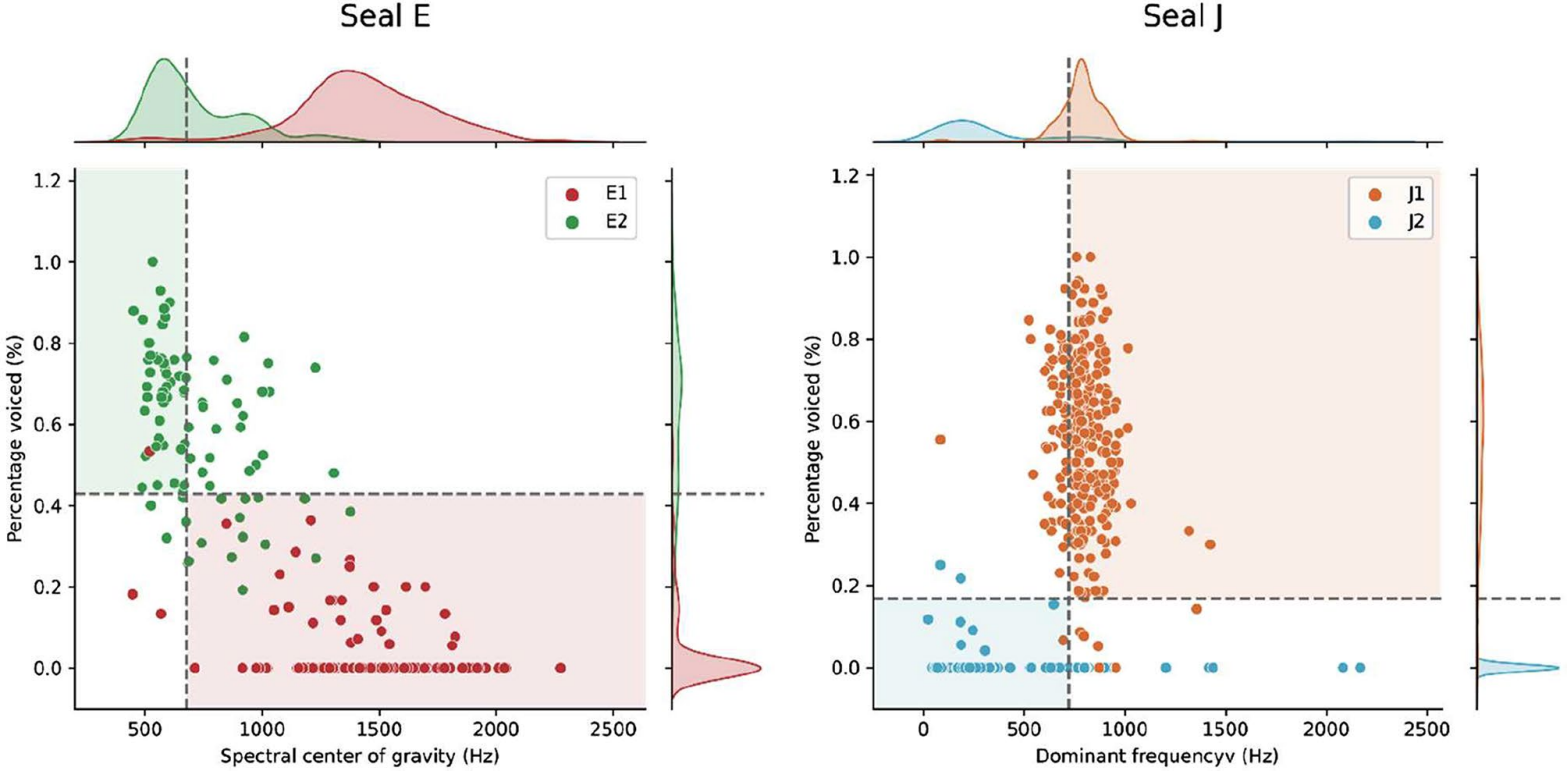
Post-experimental distribution of the distinguishing parameters. Dashed lines highlight the pre-experimental threshold used to filter out unclear stimuli and are determined as the percentile where both parameter distributions overlap (see *Stimulus selection*)

The recorded responses were acoustically and statistically compared to the pre-experimental vocalizations, to test if the two vocalization types remained acoustically distinguishable over the course of the experiment. This revealed that the distinguishing parameters, which were used to make a quantitative distinction between each seal’s respective vocalization type (see Methods section below), had slightly but statistically significantly shifted (Mann-Whitney U test, *p* < 0.0001 for all acoustic parameters, except for dominant frequency of J1, *p* < 0.05). However, the two vocalization types of both seals remained distinct and recognizable (see Figs. 2 and 9).

To quantitatively probe a potential difference between the pre-experimental vocalizations and experimental responses (see Figs. 2 and 9), we analyzed the effect of vocalization type (E1/E2 and J1/J2) and experimental stage (pre/post) on parameter values using a 2-way ANOVA [*statsmodel* Python library; 48]. This revealed that a higher amount of the acoustic parameters’ observed variance is explained by vocalization type as opposed to experimental stage, demonstrating the distinctiveness of vocalizations (see Table 1). However, more of the parameter duration’s variance is explained by experimental stage than by vocalization type (see *Duration* for further analyses).

**Table 1:** For almost all acoustic parameters, more of the observed variance is explained in a 2-way ANOVA (as given by the sum of squares) by the seals’ vocal type than by the experimental stage. Marked in bold are the distinguishing parameters (see subsections ‘Stimulus selection’ and ‘Response parameters’)

Acoustic Parameter	Seal E		Seal J	
	Sum Sq Vocal Type	Sum Sq Experimental Stage	Sum Sq Vocal Type	Sum Sq Experimental Stage
Spectral Center of Gravity	**1.31 × 10** ^**8**^	**1.90 × 10⁷**	9.36 × 10^7^	2.54 × 10^6^
Percentage Voiced	**114**	**2.85**	**114**	**0.213**
Median Harmonicity	457 × 10^2^	771	5.99 × 10^4^	9.12 × 10^3^
Dominant Frequency	6.61 × 10^6^	2.46 × 10⁷	**2.03** ** × 10** ^**8**^	**1.48** ** × 10** ^**6**^
Duration	0.785	12.4	7.05	15.1

**Fig. 3:**
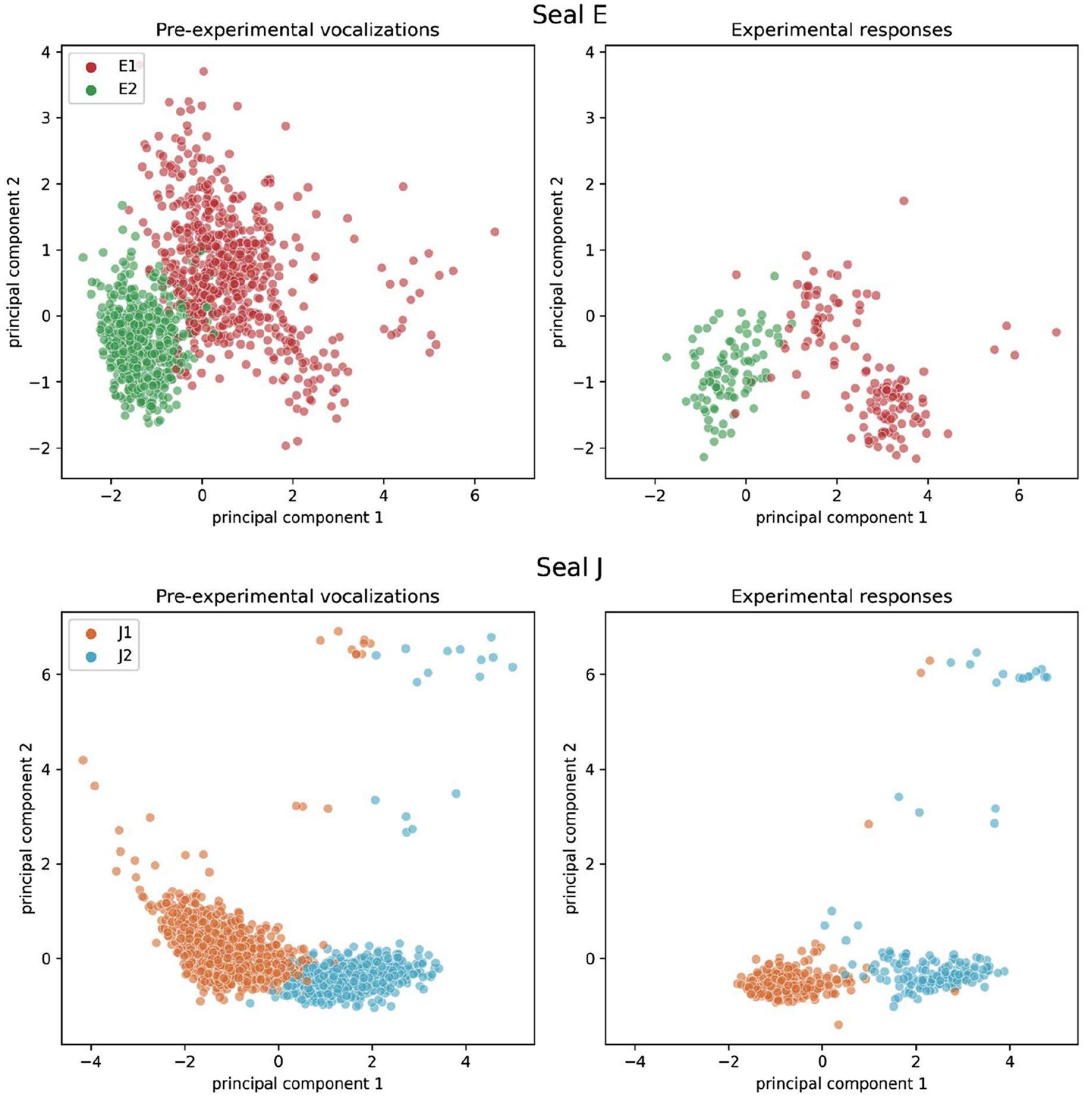
PCA visualization of the distribution of pre-experimental vocalization types (left) and the experimental responses (right). The color indicates the type of the vocalization (Type 1 and Type 2, respectively)

To visually confirm that both vocalization types could still be told apart clearly and were thus assigned correctly by the experimenter, we conducted a principal component analysis (PCA). The PCA revealed that the distribution of the investigated acoustic parameters had indeed shifted, though they did so similarly in both vocalization types (see Fig. 2). The response vocalization types appear at least equally distant as the pre-experimental vocalizations (see Fig. 3) which may explain why seals could discriminate between the vocalization types.

### Duration of vocalizations

**Fig. 4:**
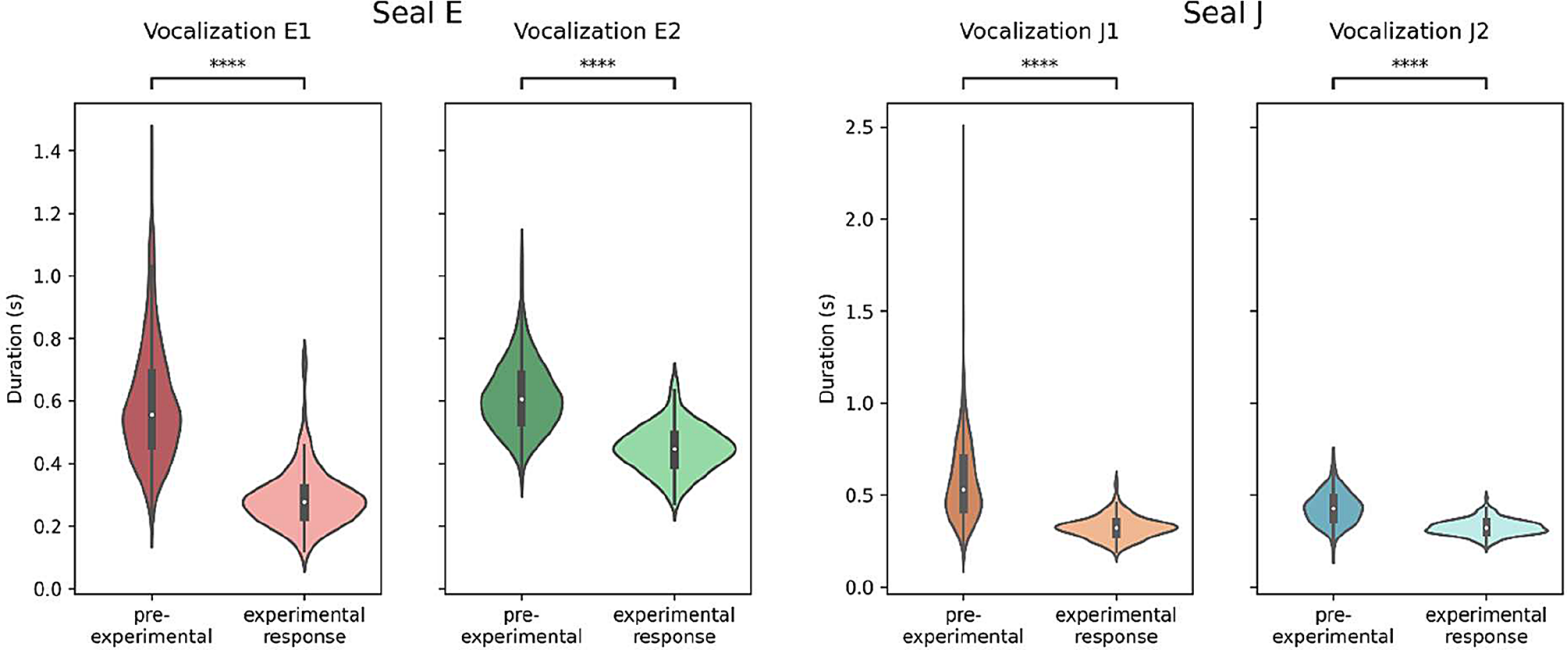
The distributions of the pre-experimental vocalizations’ (dark, ‘pre-experimental’) and responses’ (light, ‘experimental response’) duration for seal E and seal J show a significant drop in vocalization duration. Stars indicate significance level (****: *p* < 0.0001)

During the experiments and analyses, we noticed a possible drop in the responses’ duration over time. We assessed and quantified this by comparing the pre-experimental vocalizations with the response vocalizations during the experiment. The analysis revealed that the duration of seal E’s emissions decreased significantly for both vocalization types (see Fig. 4). The median duration of vocalization E1 dropped from 0.56s to 0.28s (Mann-Whitney U test, *U* = 103892.5, *p* < 0.0001); the median duration of vocalization E2 decreased from 0.61s to 0.45s (Mann-Whitney U test, *U* = 76580.5, *p* < 0.0001).

Seal J’s response duration also decreased significantly for both vocalization types (see Fig. 4). The median duration of vocalization J1 dropped from 0.53s to 0.32s (Mann-Whitney U test, *U* = 336770.5, *p* < 0.0001); the median duration of vocalization J2 decreased from 0.43s to 0.32s (Mann-Whitney U test, *U* = 158615.5, *p* < 0.0001).

## Discussion

All harbor seals learned each task in ≤ 16 sessions (Fig. 1), demonstrating vocal usage and comprehension learning under controlled conditions. Further, both seals showed the ability to generalize beyond trained stimuli. These results echo those from a grey seal who could vocally respond to trained stimuli and generalize to novel stimuli of the same call classes [23]. Qualitatively, while seal E learned all tasks equally fast (five, six and, five sessions for Experiments 1–3, respectively), seal J’s performance fluctuated considerably (four, eight, and sixteen sessions for Experiments 1–3, respectively). There are several possible explanations for this observed discrepancy in seal J’s learning times. Call matching tasks qualify as complex vocal usage learning [1, 22, 49]); perhaps our tasks imposed different cognitive demands on the two individuals. Alternatively, attentional or non-cognitive factors may have influenced their performance; anecdotally, seal E appeared more focused and eager to participate in the experiments, while seal J appeared sometimes nervous and reluctant to participate.

In *Experiment 1*, the seals successfully switched from a visual to an auditory cue, i.e., they reliably responded to novel cues from a different modality. This reflects their ability to vocally learn; in fact, the definition of vocal learning includes associating an already existing vocalization with a novel context. Considering the long training phase (see Fig. S1), this task took the longest to learn. An underlying reason may be the comparatively complex setup of *Experiment 1*. Instead of using a less demanding setup with ten identical stimuli of Type 1, and ten identical stimuli of Type 2, we used 10 instances of Type 1 stimuli and 10 instances of Type 2 stimuli. This large training set might have made the task more demanding, but served as a suitable preparation for the later following vocal generalization task [see 22 for a discussion on training set size]. Nevertheless, both seals learned to discriminate the acoustic stimuli and correctly respond to them.

In *Experiment 2*, the animals responded correctly to a combination of both trained and untrained stimuli (with replacement), within six (seal E) and eight (seal J) sessions. Both seals answered the untrained stimuli subset significantly more often correctly than incorrectly and already started with a high success rate; this suggests that they succeeded at vocal type matching, rather than having memorized individually trained stimuli and responding accordingly. This is further supported by the finding that they did not answer significantly more correctly to trained than to untrained stimuli. In other words, if the seals had memorized all trained stimuli of *Experiment 1*, we would expect the seals to perform significantly worse on the untrained stimuli, which was not the case.

*Experiment 3* showed that the seals were able to generalize beyond a trained set of stimuli. Only novel stimuli were broadcasted to the animals, each of which was played back only once during this whole experiment. Seal E reached the LC within relatively few sessions (5 sessions), while seal J needed more time to learn the task (16 sessions). Irrespective of the number of sessions needed to learn, both seals showed that they are capable of auditory generalization. This may not be surprising as the abilities to categorize and generalize are cognitively fundamental [44, 47]: they enable to organize information efficiently, to identify similarities (and/or differences) among discriminable stimuli and to form categories [50]. Our findings indicate that harbor seals learn to distinguish between auditory cues, potentially based on their similarity, and transfer this distinction to novel stimuli. Such capacities might be crucial for recognising e.g., territorial sounds, such as flipper slapping, which are an integral component of harbor seals’ agonistic interactions and territory display [51]. For example, harbor seals might use the vocalizations of opponents to identify and react to intruders of their territory [52]. The ability to distinguish between auditory cues, which we report here, is crucial for such responses.

Beyond generalization, both seals reacted differently to the two distinct vocalization types. This demonstrates vocal comprehensionlearning, i.e. learning to understand the novel “meaning” of vocalizations [1]. Not only did the seals learn to react to the broadcasted vocalizations, they also learned to respond differently depending on the type of the broadcasted vocalization. This ability was previously shown in a closely related species, the grey seal [23], where the tested seal succeeded at call generalization in an extended study of a previously failed experiment [22]. Stansbury and colleagues [23] pointed out that they used a considerably larger training set than Shapiro et al. [22], which underpinned our decision to use a relatively large training set in *Experiment 1*. The tested grey seal even generalized beyond her own vocalizations: she responded with matching call types to calls of other grey seals [53]. Unfortunately, we could not integrate this promising approach in our study, as no pair of seals in our subject pool produced overlapping vocalizations.

Upon comparison of the acoustic parameters of pre-experimental vocalizations and vocal responses (see Figs. 2 and 3, and 9), we detected some differences in acoustic parameters between pre-experimental vocalizations and experimental responses. This is not surprising, as during training some vocalizations were selectively shaped for clarity (J1) or to be distinct from others (E1 was shaped pre-experimentally to be distinct from E2 [32, 41]). Such a change in parameter composition is common when selectively shaping vocalizations [e.g., 54]. Notably, the duration of both seals’ vocal types dropped (see Fig. 4), which was explained more by experimental stage rather than vocal type (see Table 1). This could indicate an efficient strategy, where the shortening of this salient vocal feature led to the faster delivery of a food reward.

Our data demonstrate vocal contextual learning as a result of experimental training and testing. While such results provide the baseline of confirming a species’ capacity for a specific behavior, the use of this capacity in the wild remains unconfirmed. We cannot claim that harbor seals associate vocalizations with different contexts through experience in the wild, but this capacity would certainly benefit, for instance, the development of a male territorial display during the breeding season.

Future work could focus on the purported capacity for vocal production learning in harbor seals [21, 28, 31, 42]. The observed altered vocal parameter distribution in our study could indirectly point to this ability but needs to be tested under controlled conditions. While excellent respiratory and articulatory control is known for some pinniped species [19, 20, 55], little is known about harbor seals’ ability to actively adjust acoustic parameters [21]. By now, it is known that harbor seal pups are vocally plastic: they can adjust their fundamental frequency in response to noise [30]. To show vocal production learning, follow-up experiments need to test whether seals can maintain adjusted parameters over time. In particular, we suggest testing vocal production learning by disentangling the control on all three levels of sound production, i.e., their breathing, larynx, and upper vocal tract [21].

## Conclusion

Our experiments tested and quantitatively demonstrated that harbor seals are both vocal usage and comprehension learners. Beyond the ability of contextual learning, the harbor seals have shown that they are capable of auditory generalization, similar to the closely related grey seal. To map the mechanisms behind harbor seals’ vocal control, future studies should examine vocal production learning in harbor seals, comparable to the existing studies in grey seals [20, 24]. Ultimately, vocal learning research in pinnipeds will provide a valuable model of human speech evolution based on the remarkable similarities with human sound production [41, 56–58].

## Methods

### Subjects and housing

Two harbor seals participated in the study: a 19-year-old male harbor seal, Jannik, born at Zoo Duisburg (seal J), and a 9-year-old female Elektra, born at Zoo Osnabrück (seal E). The seals were housed together with five captive-born harbor seals (two of which were sent to a different zoo in September 2021) in a 230,000-liter freshwater tank and 300 m^2^ enclosure at Zoo Cleves, Germany. The seals’ training routine consisted of 30- to 60-minute-long sessions up to three times per day. Usually, one daily training session was conducted by zoo staff (medical and enrichment training); a second session was conducted by the experimenter (research training). Occasionally, research training occurred twice per day. The seals were trained year-round in most weather conditions except for heavy wind and storms. The training method was operant conditioning: correct responses were positively reinforced, whereas incorrect ones were followed by a neutral response (least-reinforcement scenario, consisting of a ~ 3 s pause). Reinforcement consisted of a variable amount and composition of four fish species: capelin (*Mallotus villosus*), herring (*Clupea harengus*), sprat (*Sprattus sprattus*), and mackerel (*Scombrus scombrus*).

### Pre-training and experimental procedure

#### General

This study tested whether harbor seals are capable of vocal usage and comprehension learning. Our approach builds on several published studies [17, 22, 23]. Through three experiments, we tested whether the seals were capable of

- vocally responding to a novel cue (vocal usage learning);
- reacting differently to auditory stimuli (vocal comprehension learning);
- generalizing stimuli.

**Fig. 5:**
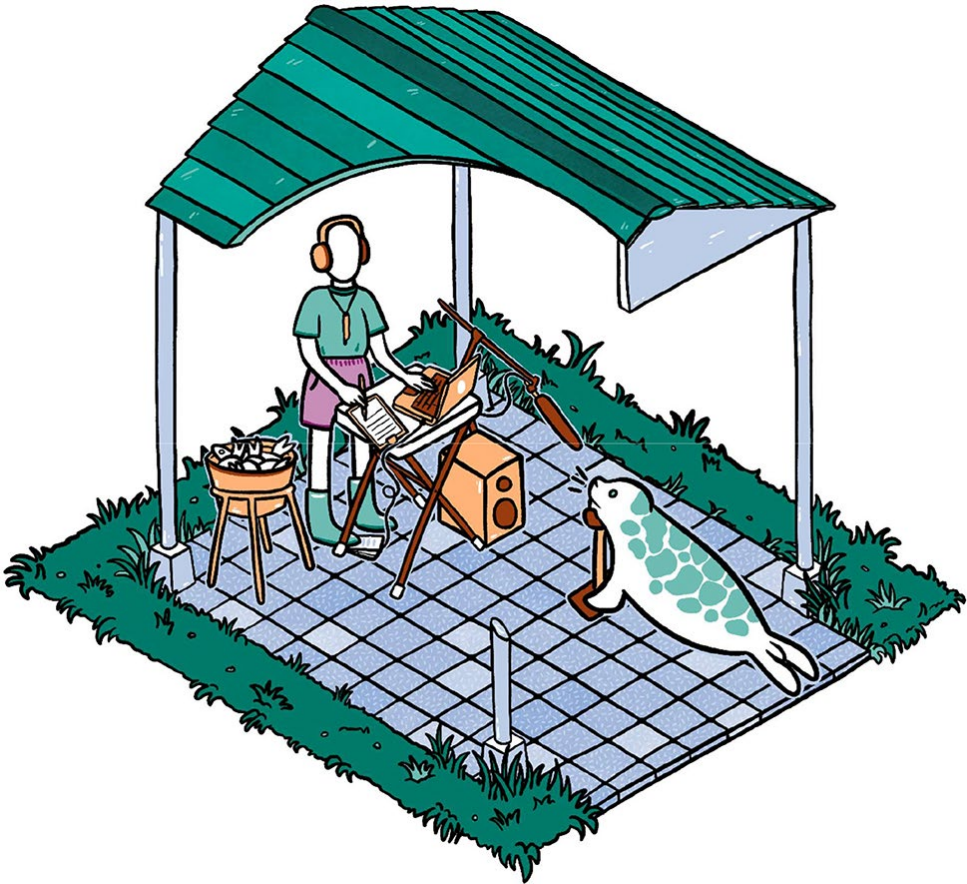
Experimental setup, with “acoustically blindfolded” experimenter (behind the laptop) and the stationed experimental animal positioned in front of the microphone and loudspeaker

**Fig. 6:**
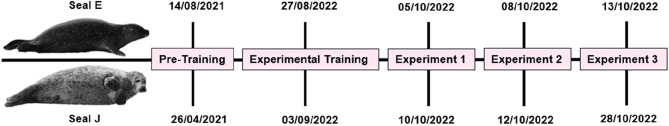
Timelines of the pre-training, experimental training and final experiments for seal E (above) and seal J (below)

The animals participating in the study were experimentally naive. Therefore, in a pre-training series, the seals were trained on basic behaviors such as stationing and targeting, but also specifically experimentally relevant behaviors, such as ‘vocalize on cue’, ‘vocalize and withhold from vocalizing’, and ‘emit two different vocalizations upon distinct cues’ [for methods and results, see 32]. The seals were trained once a day, 5–6 times/week during *Pre-training*, which was increased to up to twice a day, 5–6 times/week during the *Experiments*. *Pre-training* describes the phase during which we brought the animals to the level of being trainable for and testable on our experimental tasks; this includes the above-mentioned dedicated training and *Introduction* of the setup, auditory stimuli, and station (see Fig. 5, 6, and S5). *Experimental training* describes the training required to perform the experiments. *Experiment* describes the actual experiments. For *Pre-training*, *Experimental training*, and *Experiments*, we selected as learning criterion (LC) 80% correct choices during four consecutive sessions in each testing scenario. Only during *Experimental training*, time constraints deriving from the zoo spurred us to lower the LC from four times 80% correct choices to two times 80% correct choices after 35 (seal E) and 25 (seal J) sessions. For one session, the chance of getting 80% or more correct responses in 20 trials is < 0.006 when guessing at random. For all experiments, we calculated the statistical significance of the number of sessions within each experiment, under the null hypothesis of random guessing (i.e., if the seal would not have learned and would have a 50–50 chance rate of answering each trial correctly). To do so, we computationally calculated the exact probability of a randomly responding seal reaching the LC, 4 consecutive sessions of 80% or more correct responses, within the observed number of sessions or less (i.e., the p-value regarding the above null-hypothesis; for more details on this computation, see Supplementary Material).

#### Experimental setup

All experiments were conducted in a 300 m^2^ research enclosure, which was separated from the main enclosure by a gate. The research enclosure featured a 3 × 5 × 2.3 m carport above a 13.2 m^2^ paved platform, where all experiments took place (see Fig. 5). Before each training and testing, the experimenter would enter the main enclosure, move to the research enclosure (hereafter enclosure), and call the focal animal from the gate area.

#### Pre-training

During *Pre-training* the seals were trained to associate a vocalization with a distinct visual cue (hand sign), to vocalize or refrain from vocalizing on cue, and to associate two different vocalizations with two different visual cues [32]. Once the animals had learned to respond to two visual cues with two different vocalizations, the hand signs were faded out and replaced by auditory stimuli. For a more detailed pre-training protocol, see [32] and Supplementary Material.

#### Experimental training: shift from visual to auditory cue

**Fig. 7:**
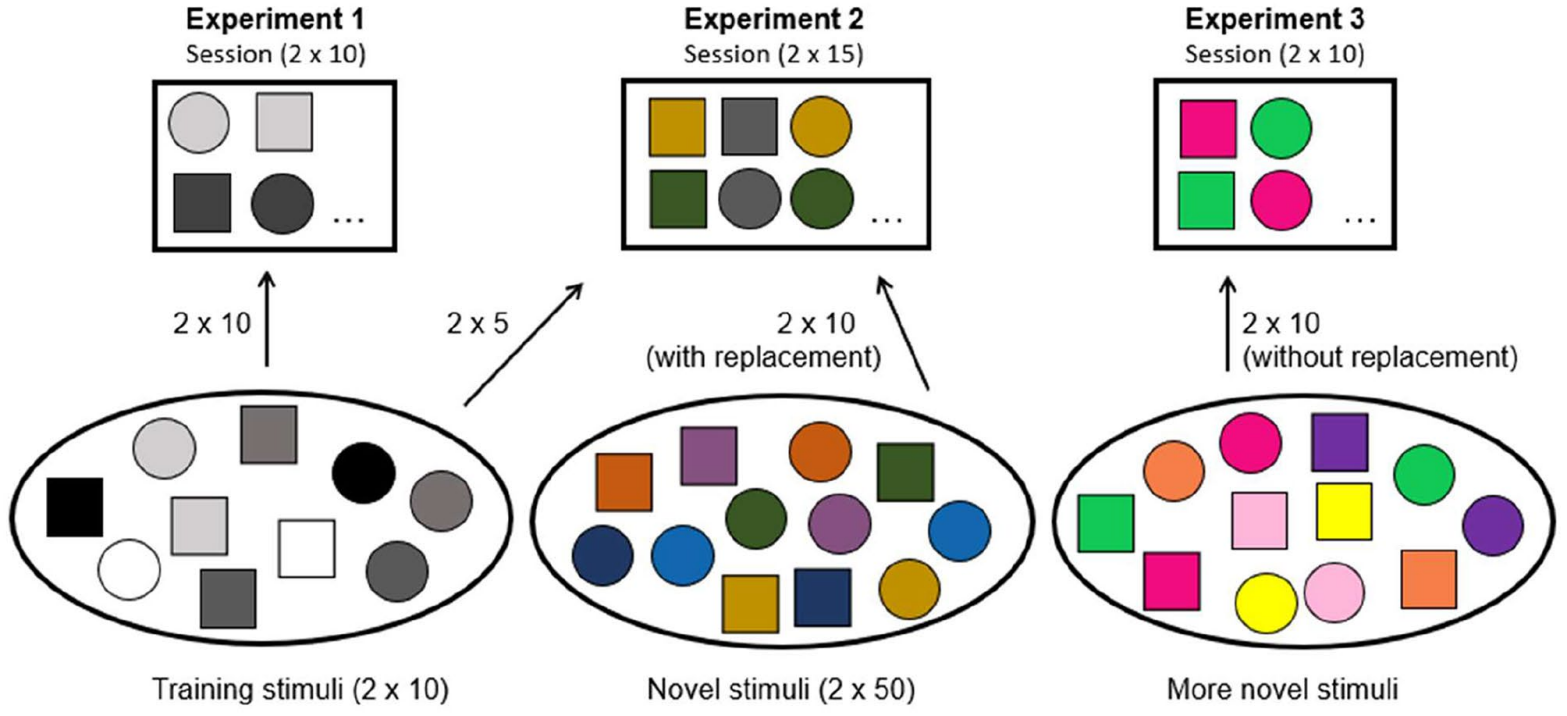
The number of novel stimuli increased in each subsequent experiment. In this visualization of how stimuli were sampled for a session in each of the experiments, the shapes indicate the vocal type (1/2). The colors indicate the “pools” from where a stimulus was sampled, where white to grey shades (left ellipse) indicate origination from the trained stimulus pool (*Experiment 1*), and chromatic shapes (middle and right ellipses) indicate untrained stimuli used in experiments 2 and 3. The “Novel stimuli” and “More novel stimuli” pools were entirely disjunct, and the stimuli of all trials during *Experiment 3* had never been heard before by the seals

**Fig. 8:**
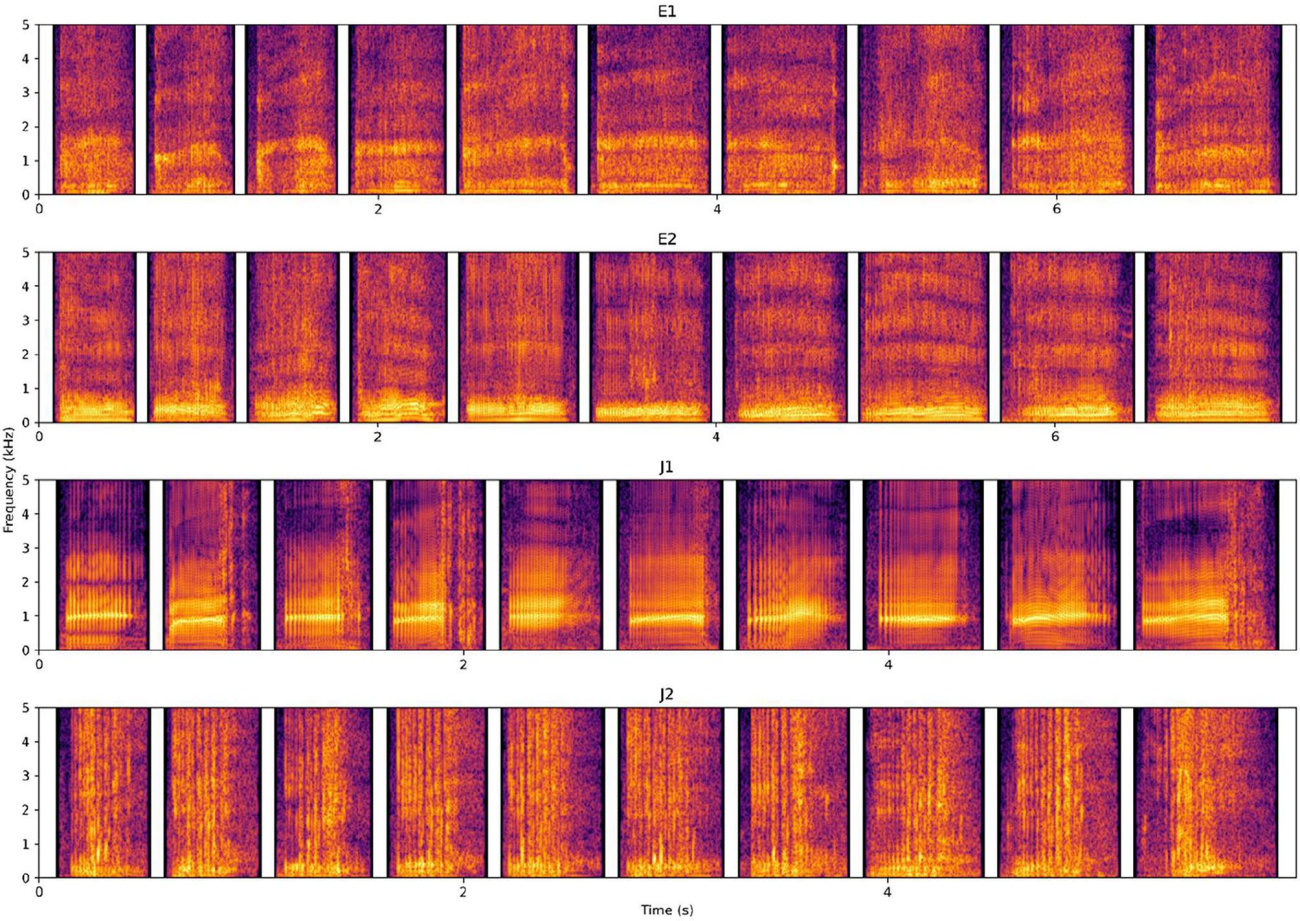
The 10 vocalizations of both types from seals E and J, used during training and in *Experiment 1*, show that the two types form two acoustically distinguishable categories. The spectrogram was obtained with the *Parselmouth* package in Python (v0.4.3, Praat 6.1.38; window length = 0.02s, dynamic range = 80 dB, max. frequency = 5 kHz [60–62])

Once the animals reliably responded with any vocalization to the playbacks, experimental training started. The seals heard 20 individual stimuli per session in pseudo-randomized order. The 20 stimuli consisted of 10 items (see Figs. 7 and 8) of vocalization Type 1, and 10 instances of vocalization Type 2. These stimuli were recordings of the seals’ own vocalizations and were pre-selected according to the steps below (see subsections *Experimental setup* and *Stimulus and session generation*). Reinforcement occurred when the animals responded to vocalization Type 1 with Type 1, or to vocalization Type 2 with Type 2, respectively. Responses were judged as correct or incorrect by the experimenter based on aural assessment and visualization of the spectrogram (see Fig. S4). After an incorrect response, the failed trial was repeated up to three times. If the animal had not responded correctly upon the third repetition, the experimenter moved on to the next trial. Any vocalization between trials resulted in a least-reinforcement scenario. Seals had to achieve a learning criterion of 80% correct choices in two consecutive sessions before they were considered ready to participate in the actual experiments.

### Experiment 1: discrimination of trained stimuli

The first experiment aimed at assessing whether the seals had learned to shift from a visual to an auditory cue and responded reliably. The seals were tested with 20 training stimuli per session (10 per vocalization type, see Figs. 7 and 8). Here, the experimental protocol and the stimuli were identical to *Experimental training*, but crucially, there were no stimulus repetitions. The LC during testing was 80% correct choices in 4 consecutive sessions.

### Experiment 2: transition from trained to untrained stimuli

In the second experiment, untrained (i.e., novel) stimuli were introduced. The focal animal had never heard these specific sounds before, except upon producing them at least six months before. We selected a subset of 100 of these vocalizations as novel stimuli. The sessions then consisted of 10 familiar stimuli (5 of each vocalization type) randomly taken from the pool of 20 of *Experiment 1*, plus 20 (10 of each vocalization type) of these new stimuli (see Fig. 7). Each session therefore contained 10 already learned, as well as 20 (potentially re-occurring) novel stimuli, resulting in 30 trials/session. The learning criterion was kept at 4 × 80% correct responses.

### Experiment 3: generalization of novel stimuli

To test whether the seals would generalize to novel vocalizations of the same type, both vocalization types were sampled without replacement in 20 trials/session (see Fig. 7). These individual stimuli were never heard by the seals before, except upon their production (at least six months before their playback) and were removed from the pool of samples after they were used in a session. Stimuli were sampled from the same stimulus pool as described below (see *Stimulus and session generation*), after any stimuli of *Experiment 1* and *Experiment 2* had been excluded. This way we ensured that all stimuli were entirely novel and used only once, enabling testing for stimulus generalization.

### Recordings

All experiments were video recorded using a Canon Legria HF25 camcorder, as were some pre-experimental and experimental training sessions. We performed two types of audio recordings. The first, baseline recordings, were recorded between August 2021 and April 2022, i.e., before and during the pre-experimental phase (*Pre-Training and Introduction*), and later served as stimuli. The second, response recordings, consists of the animals’ vocalizations in response to the stimuli during experiments 1–3 (October and November 2022). The seals were recorded with a Zoom H6 digital recorder connected to a Sennheiser ME-67 unidirectional microphone (frequency response of 40–20.000 Hz +- 2.5 dB) covered by a foam windshield. During pre-training the microphone was handheld by the trainer; during training and testing the microphone was stationary on a tripod. In both cases, the microphone was directed at the seal at approximately 1 m.

### Playback procedure

Stimuli were played back via a Yamaha HS6 loudspeaker, connected to a Panasonic laptop over a 3.5 mm jack-to-XLR connector (see Fig. 5). Stimuli playback was controlled via a custom-made vocal usage learning application (see Fig. S4 and S5). The application, running on the experimental laptop, was designed to facilitate a double-blind study and avoid the Clever Hans effect [59]. This effect refers to a situation where an experimenter unintentionally cues an animal, which can result in the animal learning a task through cues unintended for the study’s purpose. A double-blind approach helps to avoid such inadvertent falsification. Here, by means of the custom application, stimuli were played back over the connected loudspeaker, without the experimenter knowing which stimulus was broadcasted. To achieve this, the experimenter was “acoustically blindfolded”: While the stimulus was played to the focal seal the experimenter listened to white noise over headphones (Sennheiser HD 200) so that they would not know which sound was being played until the playback was over and the seal had responded. After stimulus playback, the white noise stopped, and the experimenter could hear the seals’ response and compare the played-back stimulus type and the response’s spectrogram on the screen. Responses were assessed for correctness aurally and visually, based on comparing the heard vocalization and its spectrogram’s spectral properties to those of the played back stimulus (see Fig. S4). In order to ensure the experimenter’s ability to correctly discriminate between vocal types, the experimenter was tested prior to the start of the experiment on their discrimination abilities, which resulted in a 100% success rate. A manual assessment during the sessions was therefore sufficient and less sensitive to the background noises in the Zoo setting than a fully-automatic system.

### Combining data from experiment notes, audio, and video

During the experiment, the experimenter noted the results (correct/incorrect response) on a custom sheet for each session and seal. Before statistical analysis, the responses were revised and potentially corrected by means of the audio and video recordings, to ensure the faithful assignment of correct/incorrect responses (see subsection *Experimenter reliability* in Supplementary Material). During the three experiments no stimuli were repeated, except for a few special cases; a trial’s stimulus was repeated when (i) the seal failed to respond (e.g., due to disturbances by zoo personnel or visitors), or (ii) the seal’s response was not understandable (e.g., due to high background noise or interruptions). Such cases occurred in 4.7% and 6.6% of all trials, for seals E and J respectively, and were unpreventable due to the zoological setting of the study. A potential influence of the repetitions on the results was tested (see subsection *Repetitions* in Supplementary Material). Some factors (e.g., interruptions, novel inhabitants in the neighbor enclosure) caused sessions to be terminated (i.e., the animal left the experimental setup and did not return within the following 10 min). These sessions were not considered during data analysis. An overall number of 5.2% of sessions were terminated.

### Stimulus and session generation

#### Data collection

As a first step to preparing the experimental stimuli, pre-experimental audio recordings were manually annotated (Fourier window size: 0.05s, dynamic range: 70dB, temporal resolution 1000 time steps) using *Praat v6.1.40* [60] and each annotated vocalization was assigned the respective vocalization type. We assessed the acoustic quality based on the visual clarity of the spectrogram of the vocalization or the presence of overlapping sounds; we excluded vocalizations that had insufficient quality, mostly due to background noise. Such noise usually resulted from disturbances by talking visitors, zoo personnel, construction works, bird vocalizations, or airplanes. Seal E’s vocalization types were termed E1 and E2, and seal J’s types were termed J1 and J2 (as used throughout the manuscript), all of which are aurally and spectrally clearly distinguishable (see Fig. 8, and WAVE files in the online supplement).

#### Stimulus selection

The annotated vocalizations and relevant acoustic parameters were batch-extracted with the *Parselmouth* package [a Python library for Praat, v.0.4.1; 60, 61] in Python. We chose commonly assessed parameters of harbor seal calls, which we expected to be relevant in distinguishing the different vocalization types. These were duration, spectral center of gravity, dominant frequency, percentage voiced, and median harmonicity [61, 63, 64] (see Table 2 for extraction procedure). These measured parameters’ distributions were then compared between vocalization types (see Figs. S2 & S3 for the respective scatterplots).

**Table 2:** Extraction procedure of the acoustic parameters

Parameter	Definition
Duration (s)	Temporal interval from the start to the end of the vocalization.
Spectral center of gravity (Hz)	The weighted average frequency of the vocalization’s spectrum. The spectrum was calculated with Praat’s “*Sound: To Spectrum*” (default parameters). Then, the center of gravity was calculated using “Spectrum: Get center of gravity” (default parameters).
Percentage voiced (%)	The pitch was calculated with Praat’s “*Sound: To Pitch*” (default parameters, except for pitch_floor = 50, pitch_ceiling = 400). The number of voiced frames was divided by the number of all frames.
Median harmonicity (dB)	Harmonicity was calculated with Praat’s “*Sound: To Harmonicity (cc)*” (default parameters, except for minimum_pitch = 50). After this the median of the harmonicity values were calculated.
Dominant frequency (Hz)	The spectrum was calculated with Praat’s “*Sound: To Spectrum*” (default parameters). Then, we selected the frequency bin with the highest power.

**Fig. 9:**
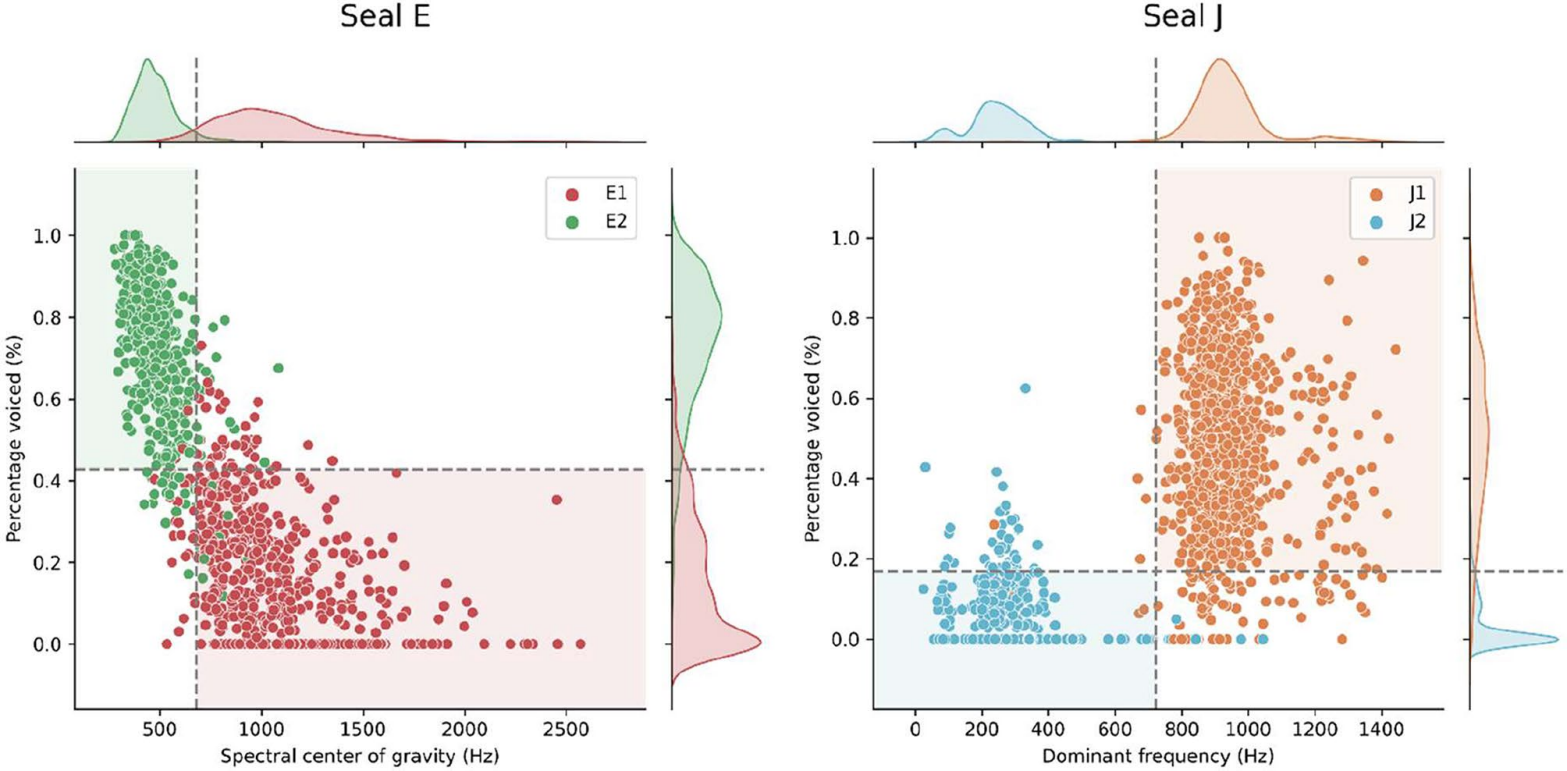
Distinct acoustic parameter values of the pre-experimental vocal repertoire of seal E (left), and seal J (right). The dashed lines represent the threshold determined based on the equal percentile of parameter distribution (seal E; 677.32 Hz center of gravity and 0.43% percentage voiced, seal J; 722.7 Hz dominant frequency and 0.17% percentage voiced). Extracted vocalizations with parameters in the matching shaded regions were retained as stimuli for training and experiments

Next, we tested which of these parameters’ distribution provided the most distinction between the two vocalization types per seal. Based on the initial dataset of extracted parameters (seal E: *N* = 670 vocalizations, 386 E1, 284 E2; seal J: *N* = 1198 vocalizations, 756 J1, 442 J2), we calculated mutual information between the parameter and the vocalization type [using *scikit-learn*’s mutual_info_classif function, 65]. For seal E, *spectral center of gravity* and *percentage voiced* were the two parameters with the highest estimated mutual information regarding vocal type (see Table S2); both parameters are significantly different between types (Mann-Whitney U tests, *U* = 343458, *p* < 0.0001 and *U* = 4935, *p* < 0.0001, respectively). For seal J, the two most distinguishing parameters were *dominant frequency* and *percentage voiced* (see Table S2), both significantly different (Mann-Whitney U tests, *U* = 709964, *p* < 0.0001 and *U* = 696220.5, *p* < 0.0001, respectively). We used these two parameters to make a quantitative distinction between each seal’s respective vocalization types, as opposed to the commonly used, but less precise aural and visual inspection. To maximize the distinctiveness of presented stimuli, we only used vocalizations from the two types where their parameter’s distributions did not overlap. For example, Seal E’s E1 vocalization had an average higher spectral center of gravity (CoG) than the E2 vocalizations (see Fig. 9). Therefore, we calculated the percentile and threshold value *p*_*eq*_ such that the *p*_*eq*_ percentile of the E2 vocalizations CoG distribution equals the *(1 – p*_*eq*_*th)* percentile of the distribution E1 vocalizations (in this case *p*_*eq*_*≈* 0.9497, corresponding to a value of 677.32 Hz, see Fig. 9). In this example, we then only used E2 with a CoG below this threshold, and all E1 with a CoG above. This ensured that only clearly distinguishable vocalizations were used as stimuli. To ensure high quality stimuli, all stimulus candidates were again aurally assessed to discard those containing non-seal sounds (see description of background noise above).

Finally, for both seals, duration was the least distinguishing vocal parameter; the vocalization types’ distributions for duration had a substantial amount of overlap (see Supplementary Material, Figure S2 and S3). This indicated that duration most likely did not play a crucial role in distinguishing E1/E2 or J1/J2 vocalizations. When selecting stimuli for the experiments, we prepared the sessions with equally distributed durations for each vocalization type (see below).

#### Session sampling

To avoid that the seals would discriminate the stimuli based on intensity, all stimuli were normalized to the same intensity via Praat’s *Sound: Scale intensity*, equalizing the root-mean-square amplitude of the stimuli [61, 62, 66]. Additionally, to exclude possible discrimination based on duration, stimuli were sampled in pairs, one stimulus per vocalization type, in such a way that the duration of paired stimuli differed by less than 0.01 s. Sessions were sampled from the different stimulus pools, which were used for *Experiment 1–3*, as described earlier (see subsections *Experiment 1–3*). Finally, the order of stimulus presentation within each session was randomized according to the Gellermann series [67] using a pre-release version of the *PyGellermann* Python package [68].

## Electronic supplementary material

Below is the link to the electronic supplementary material.

Supplementary Material 1

Supplementary Material 2

## Data Availability

The datasets used and/or analysed during the current study are available from the corresponding author on reasonable request.

## Abbreviations

Not: applicable
