# Supplementary material for: Vocal usage learning and vocal comprehension learning in harbor seals
Source: BMC Neurosci. 2024 Oct 4;25:48. doi: 10.1186/s12868-024-00899-4 (PMC11451073; doi:10.1186/s12868-024-00899-4)
Supplement: Supplementary file 1 — Supplementary Material 1 [file 12868_2024_899_MOESM1_ESM.docx]

**Supplementary Material**

**Table S1.** Extracted acoustic parameters, mean, and standard deviation values for each respective seal’s vocalization type. the chosen distinguishing parameters (see main text and Table S2) are marked in bold.

| **‍** | Seal E | | Seal J | |
| --- | --- | --- | --- | --- |
| **Acoustic parameter** | E1 | E2 | J1 | J2 |
| Duration (s) | 0.59 ± 0.18 | 0.61 ± 0.11 | 0.58 ± 0.22 | 0.43 ± 0.09 |
| Centre of gravity (Hz) | **1067.97 ± 308.96** | **476.02± 106.45** | 1102.19 ± 143.33 | 1373.78 ± 464.41 |
| Percentage voiced (%) | **0.16 ± 0.16** | **0.73 ± 0.16** | **0.49 ± 0.20** | **0.04 ± 0.08** |
| Median harmonicity (dB) | -6.55 ± 34.11 | 4.86 ± 2.26 | 1.39 ± 18.74 | -6.16 ± 27.82 |
| Dominant frequency (Hz) | 371.46 ± 384.76 | 310.93 ± 117.54 | **937.87 ± 117.53** | **257.73 ± 119.13** |

**Table S2.** Estimated mutual information between each extracted acoustic parameter and the classification of the two different vocalization types selected the distinguishing parameters between each seal’s two vocalization types. As *scikit-learn*’s calculation of mutual information for continuous variables results in an estimate and involves a minor amount of randomness^^[[1]](#footnote-1)^^, we also calculated another, deterministic measure: maximum information gain for all possible thresholds one-dimensional range of the acoustic parameter’s values (i.e., the difference in average Shannon entropy before and after splitting). Both (theoretically strongly related) measures agree on which two acoustic parameters provide the most information about the vocalization type.

|  | Seal E | | Seal J | |
| --- | --- | --- | --- | --- |
| **‍Acoustic parameter** | Mutual information | Maximum information gain | Mutual information | Maximum information gain |
| Duration (s) | 0.00661 | 0.0180 | 0.163 | 0.140 |
| Centre of gravity (Hz) | **0.540** | **0.490** | 0.248 | 0.126 |
| Percentage voiced (%) | **0.533** | **0.490** | **0.450** | **0.410** |
| Median harmonicity (dB) | 0.515 | 0.473 | 0.314 | 0.256 |
| Dominant frequency (Hz) | 0.173 | 0.0391 | **0.608** | **0.599** |


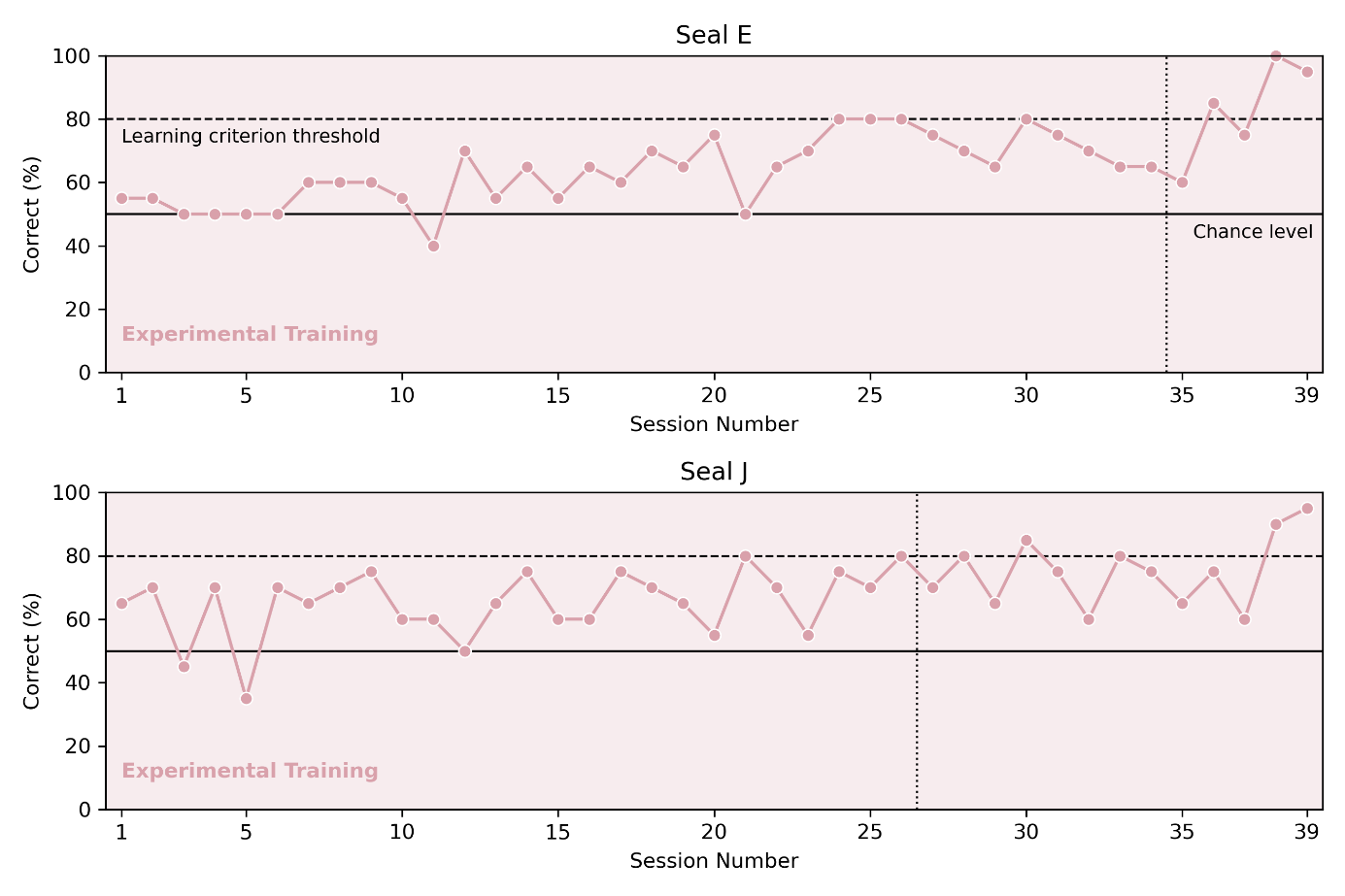


**Fig. S1**: The learning curve of seal E (above) and seal J (below), showing the increasing performance over the course of the training for experiment 1. The y-axis depicts the correct choices in %, the x-axis shows the number of sessions. The solid line represents the 50% mark on the y-axis, by which sessions would be resolved at random, while the dashed line at 80% of the y-axis represents the threshold to the learning criterion. Note that the learning criterion was reached twice, as it was reduced from 4 x 80% correct choices to 2 x 80% correct choices in consecutive sessions (marked by the dashed vertical line on the y-axis after 34 (seal E), and 26 (seal J) sessions).


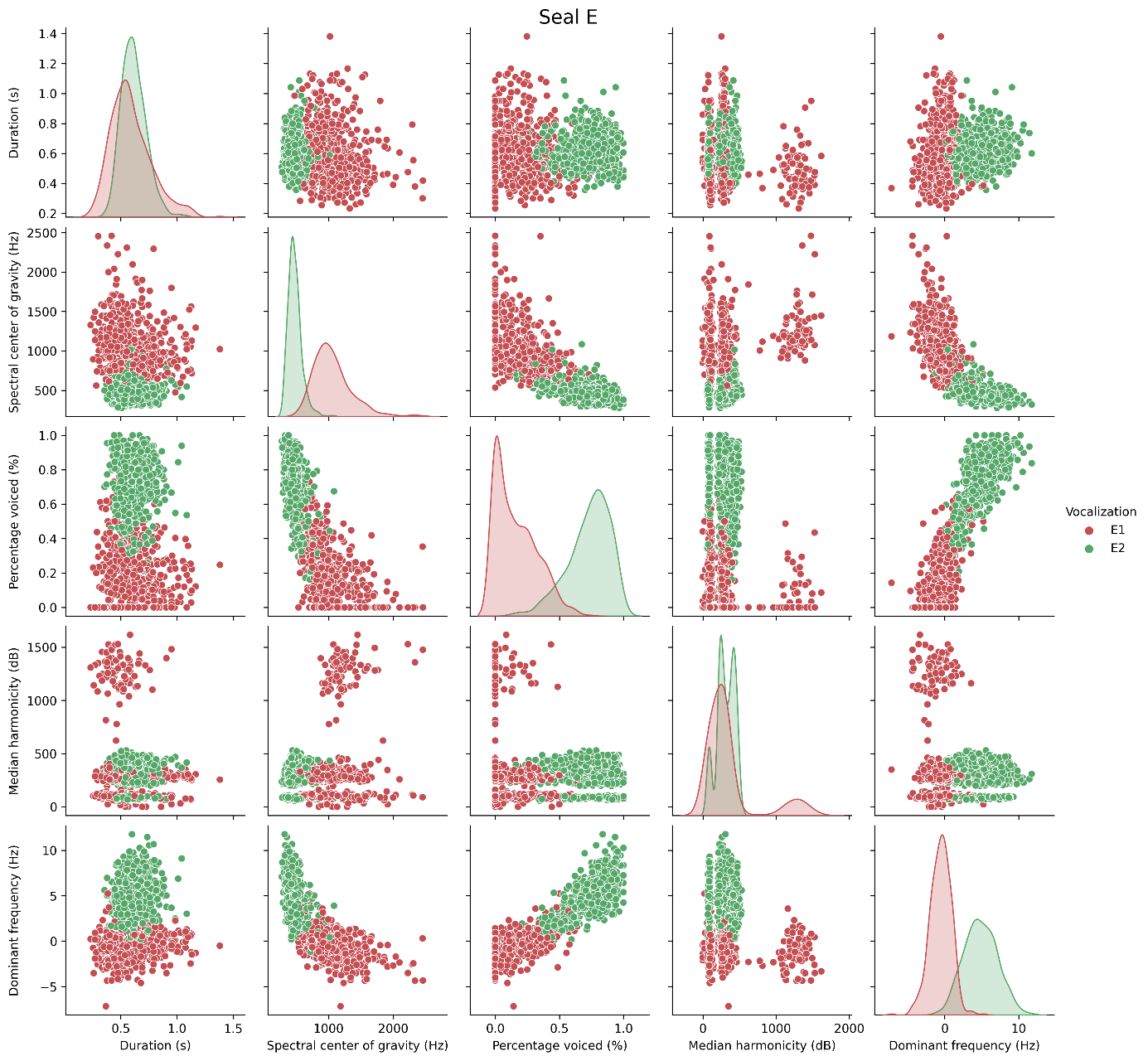


**Fig. S2:** Scatterplots of the acoustic parameter distribution of the vocalizations E1 and E2 by seal E. Note that the parameter ‘median harmonicity’ was filtered, so that it only shows values above -50 dB.


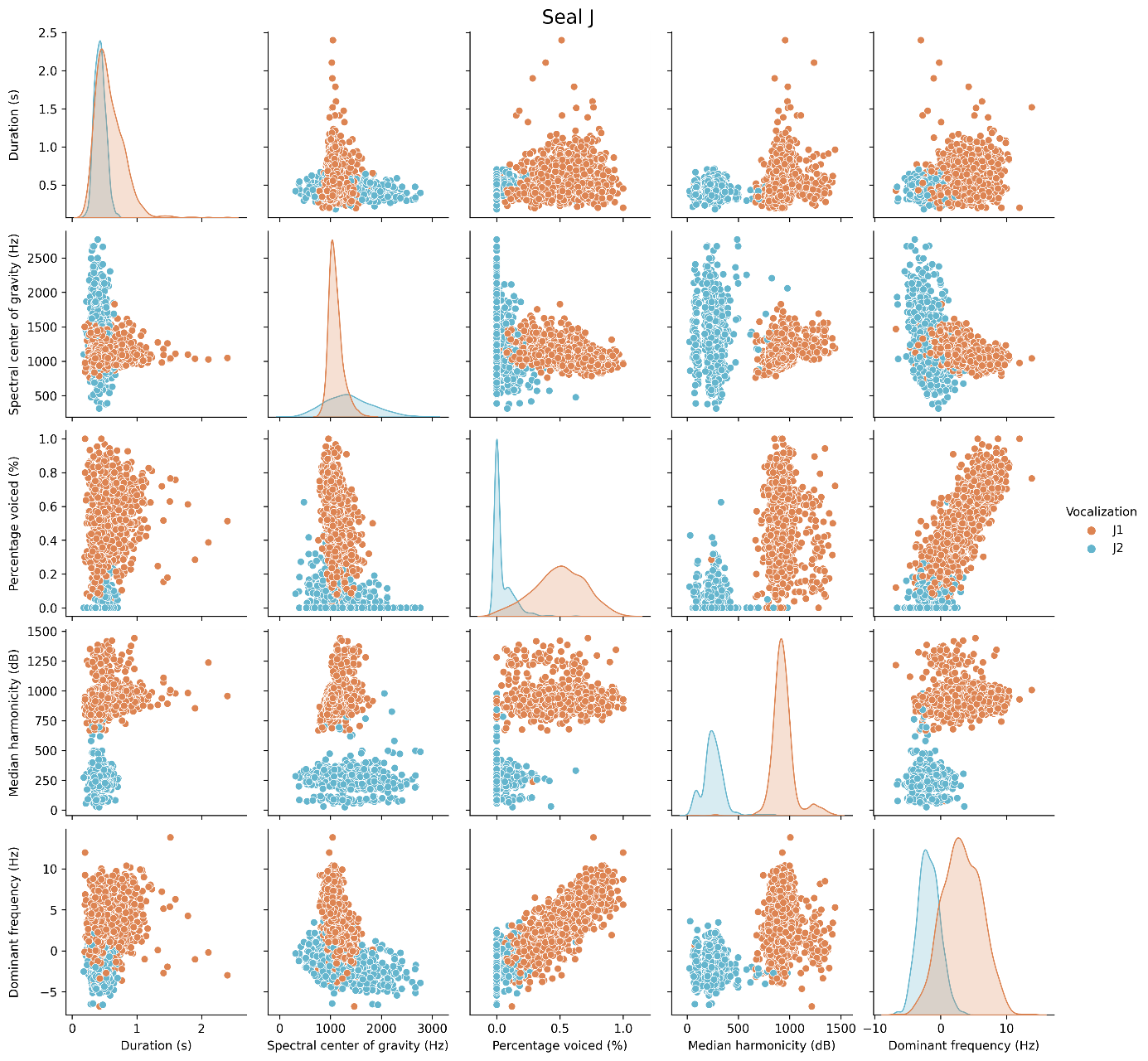


**Fig. S3:** Scatterplots of the acoustic parameter distribution of the vocalizations J1 and J2 by seal J. Note that the parameter ‘median harmonicity’ was filtered so that it only shows values above -50 dB.


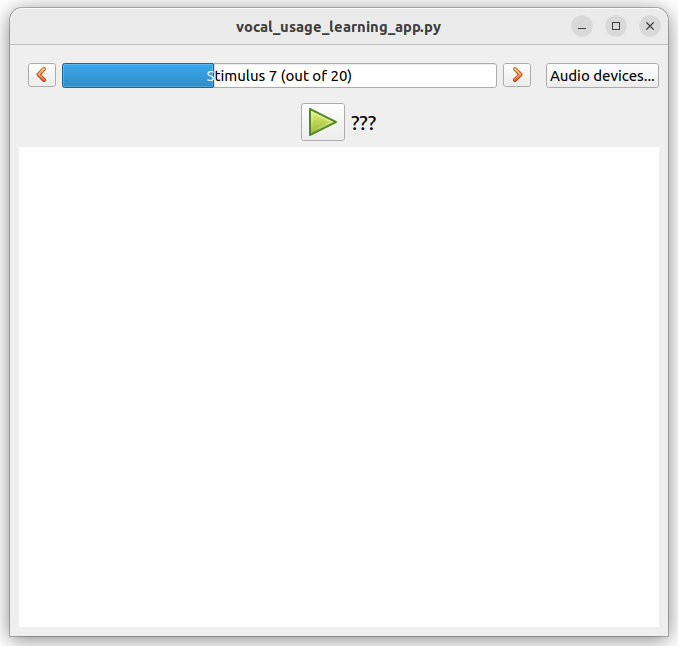

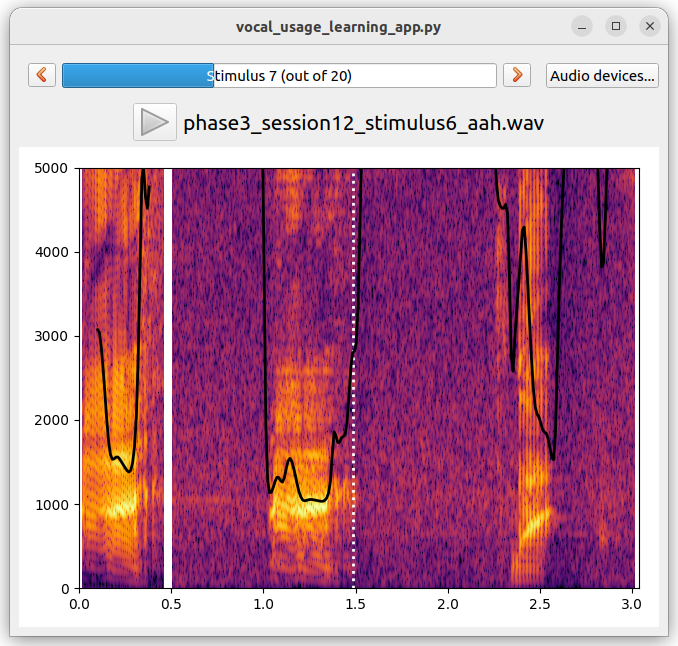


**Fig. S4**: User Interface of the custom-made vocal usage learning application. The blindfolded interface shows the progress of the experiment (left), while the response assessment window (right) facilitates the visual comparison of stimulus and response.

**Description of Pre-Experimental Training**

As the seals were experimentally naive they required pre-training before the experiments . This included training the seals to ‘target’ (push the muzzle to a specific object), ‘station’ (rest the head on a station, see Fig. 5 in the main manuscript), and ‘come’ (approach and/or follow the trainer). The conditions of vocal training developed gradually over the first weeks: Initially, animals were trained for as long as they were motivated, which resulted in a varied trial number per session for *Pre-Training A* *and B*. From then on, a standard of 20 trials per session was introduced. Note that during *Pre-Training A* no learning criterion was applied, as at this time testing under controlled conditions was, for the lack of space to single out seals, not possible.

*Pre-Training A: Vocalize On Cue*

Through operant conditioning, te seals were trained to associate a vocalization with a distinct visual cue (hand sign). Whenever the animal would vocalize, the experimenter raised their hand, and rewarded the animal, eventually resulting in a vocalization *following* the hand sign. Once the animal reliably responded to the hand sign with a vocalization, the experimenter moved on to *Pre-Training B*.

*Pre-Training B: Refrain From Calling*

The animals were trained to vocalize or remain quiet on command. For this, a visual cue for “quiet” was introduced. Sessions consisted of 20-50 trials; the exact trial number depended on the seal’s motivation. At this stage, a learning criterion (LC) of 80% correct choices in four consecutive sessions was introduced, and the training ended once the LC was reached. The order of stimulus presentation within each session was randomized according to Gellermann series (Gellermann, 1933).

*Pre-Training C: Emit Two Distinct Vocalizations*

Seal J offered two different vocalizations, which were associated with two separate cues (individual hand signs). Seal E was actively taught a second vocalization type, by shaping the original vocalization: whenever her vocalization varied notably, seal E was rewarded. This continued until the novel vocalization was aurally distinguishable from the original and was then associated with a visual cue (a different hand sign). In 20 trials/session, with an LC of 4 x 80% correct choices in consecutive sessions, it was tested whether the seals could emit distinct vocalizations upon presentation of the individual hand signs. The order of stimulus presentation within each session was randomized according to Gellermann series (Gellermann, 1933)

*Introduction: Experiment Preparation*

The animals were habituated to the novel setup, i.e., to position their chin on top of an anchored station, with the loudspeaker and computer/table in front of them (see Fig. 5 in the main manuscript). The seals were then trained to respond vocally to auditory stimuli, which consisted of their own pre-recorded vocalizations (see *Stimulus Generation* in the Methods section). This was achieved by pairing the visual cue (hand sign) with the auditory cue (playback), and successively fading out the visual cue. Once the animals reliably responded to the playback stimuli, training of *Experiment 1* started.


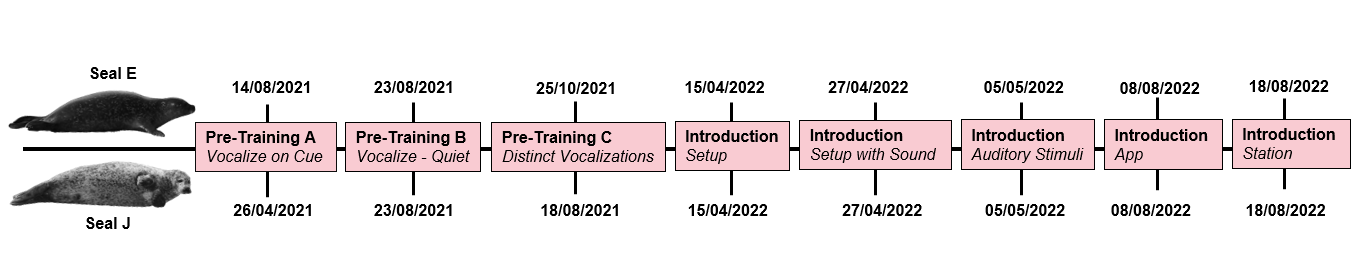


**Fig. S5:** Overview *Pre-Training A-C*, which includes the Introduction. *Introduction* refers to all training that did not need dedicated sessions but was more variable as opposed to *Pre-Training A-C*.

# Significance of reaching the learning criterion and p-value calculations

To assess the statistical significance of the seals reaching the learning criterion (LC) of 4 consecutive sessions with 80% or more correct responses, we calculated the exact p-value for the seals to reach this LC in the observed number of sessions.

Under the assumed null hypothesis, the probability distribution of the number of correct responses follows a binomial distribution $B\left( n,0.5 \right)$ with $n$ the number of trials per sessions (i.e., $n=20$ for experiments 1 and 3, and $n=30$ for experiment 2).

As a result, the chance of successfully reaching the 80% threshold in a single session equals $p_{1}=\sum_{i=16}^{20} \left( 20 i \right){0.5}^{20}\approx0.00591$ in experiments 1 and 3; for experiment 2, $p_{1}=\sum_{i=24}^{30} \left( 30 i \right){0.5}^{30}\approx0.000162$.

Next, we define $\underline{p_{4}}\left( n,k \right)$ as the probability of a sequence of $n$ sessions and ending on $i$ successful sessions while not containing 4 sequential successes in a row. $\underline{p_{4}}\left( n,k \right)$ can be calculated recursively as follows:

$$\underline{p_{4}}\left( n,k \right)=\{1 if n=0 and k=0 0 if n=0 and 0<k<4 \left( 1-p_{1} \right)\cdot\sum_{i=0}^{3} \underline{p_{4}}\left( n-1,i \right) if n>0 and k=0 p_{1}\cdot\underline{p_{4}}\left( n-1,k-1 \right) if n>0 and 0<k<4$$

Based on this, the probability of reaching the 4 x 80% LC in exactly $N$ sessions can be calculated as the probability of $N-4$ sessions without 4 consecutive successes and ending on a failed session, followed by 4 successful sessions: $p_{4}\left( N \right)=\underline{p_{4}}\left( N-4,0 \right)\cdot p_{1}^{4}$.

Finally, the desired p-value is a simple sum of the probabilities of reaching the LC in $N$ sessions or fewer: $p=\sum_{i=4}^{N} p_{4}\left( N \right)$. Table S2 shows the calculated p-values for both seals in all three experiments.

**Table S2:** Calculated p-values for both seals in all experiments

|  |  | Sessions (*N*) | p-value |
| --- | --- | --- | --- |
| Experiment 1  (20 trials/session) | Seal E | 5 | $2.43\times{10}^{-9}$ |
|  | Seal J | 4 | $1.22\times{10}^{-9}$ |
| Experiment 2  (30 trials/session) | Seal E | 6 | $7.86\times{10}^{-13}$ |
|  | Seal J | 8 | $1.31\times{10}^{-12}$ |
| Experiment 3  (20 trials/session) | Seal E | 5 | $2.43\times{10}^{-9}$ |
|  | Seal J | 16 | $1.58\times{10}^{-8}$ |

**Experimenter Reliability**

We assessed the experimenter’s decision reliability by calculating the correlation of notes taken during the experiment and the post-experimental annotations (performed by the same person). The high correlation (Pearson’s r, r=0.941, *p*<0.0001) shows a high experimenter reliability.

**Repetitions**

During the experiment, in certain contexts, stimuli had to be repeated (see *Combining data from experiment notes, audio, and video*in the Methods). Whether or not a trial was repeated did not have a significant influence on the seals’ performance (Fisher’s exact test, prior odds ratio=0.806, *p*>0.05).

1. The values reported are obtained by calling *scikit-learn*’s mutual_info_classif function with a default value for n_neighbors=3 and a fixed random seed, random_state=42. Other random seeds resulted in slightly different values but agreed on the parameter ranking based on mutual information with the vocalization type. See <https://scikit-learn.org/stable/modules/generated/sklearn.feature_selection.mutual_info_classif.html> for more details on mutual_info_classif and the method used to calculate the reported values. [↑](#footnote-ref-1)
